# Podocalyxin promotes proliferation and survival in mature B-cell non-Hodgkin lymphoma cells

**DOI:** 10.18632/oncotarget.21283

**Published:** 2017-09-27

**Authors:** Estíbaliz Tamayo-Orbegozo, Laura Amo, Marta Riñón, Naiara Nieto, Elena Amutio, Natalia Maruri, Miren Solaun, Arantza Arrieta, Susana Larrucea

**Affiliations:** ^1^ Regulation of the Immune System Group, BioCruces Health Research Institute, Hospital Universitario Cruces, Barakaldo, Bizkaia, Spain; ^2^ Cell Culture Unit, BioCruces Health Research Institute, Hospital Universitario Cruces, Barakaldo, Bizkaia, Spain; ^3^ Hematology and Hemotherapy Service, BioCruces Health Research Institute, Hospital Universitario Cruces, Barakaldo, Bizkaia, Spain; ^4^ Flow Cytometry Unit, BioCruces Health Research Institute, Hospital Universitario Cruces, Barakaldo, Bizkaia, Spain

**Keywords:** podocalyxin, lymphomagenesis, obinutuzumab resistance, glutaminolysis, pentose phosphate pathway

## Abstract

Podocalyxin (PCLP1) is a CD34-related sialomucin expressed by some normal cells and a variety of malignant tumors, including leukemia, and associated with the most aggressive cancers and poor clinical outcome. PCLP1 increases breast tumor growth, migration and invasion; however, its role in hematologic malignancies still remains undetermined. The purpose of this study was to investigate the expression and function of PCLP1 in mature B-cell lymphoma cells. We found that overexpression of PCLP1 significantly increases proliferation, cell-to-cell interaction, clonogenicity, and migration of B-cell lymphoma cells. Furthermore, PCLP1 overexpression results in higher resistance to death induced by dexamethasone, reactive oxygen species and type II anti-CD20 monoclonal antibody obinutuzumab. Strikingly, enforced expression of PCLP1 enhances lipid droplet formation as well as pentose phosphate pathway and glutamine dependence, indicative of metabolic reprogramming necessary to support the abnormal proliferation rate of tumor cells. Flow cytometry analysis revealed augmented levels of PCLP1 in malignant cells from some patients with mature B-cell lymphoma compared to their normal B-cell counterparts. In summary, our results demonstrate that PCLP1 contributes to proliferation and survival of mature B-cell lymphoma cells, suggesting that PCLP1 may promote lymphomagenesis and represents a therapeutic target for the treatment of B-cell lymphomas.

## INTRODUCTION

Podocalyxin (PCLP1), also known as podocalyxin-like protein 1, PODXL, PCLP, PC, or Gp135, is a CD34-related sialomucin expressed in renal podocytes, vascular endothelia, platelets, a subset of neurons, haematopoietic progenitors, embryonic stem cells and various types of cancer [1]. PCLP1 expression is repressed by p53 and positively regulated by Wilms’ tumor suppressor-1 [2]. Through its association with actin cytoskeleton, PCLP1 regulates cellular morphology and adhesion, exhibiting both pro-adhesive and anti-adhesive properties depending on cellular context [3, 4]. In high endothelial venules, PCLP1 acts as a pro-adhesive molecule which binds to lymphocyte L-selectin during the homing process [4]. High expression of PCLP1 has been associated with an aggressive phenotype and unfavorable prognosis in breast, colorectal, prostate, ovarian and renal cancer [5–9]. In MCF7 breast cancer cells, PCLP1 enhances migration, invasion, and matrix metalloproteinase expression as well as activation of mitogen-activated protein kinase (MAPK) and phosphoinositide-3 kinase (PI3K), both pathways involved in proliferation, survival and migration [10]. Moreover, PCLP1 regulates transforming growth factor-β-induced epithelial-mesenchymal transition, a mechanism endowing epithelial cells with migratory and invasive capabilities [11]. Recent reports highlight the participation of PCLP1 in chemoresistance to cisplatin [12, 13]. The expression of PCLP1 has been detected in blasts from the majority of acute myeloid leukemia, acute lymphoblastic leukemia and cutaneous myeloid sarcoma patients [14], although the functional significance of this protein in the progression of haematological malignancies and its expression in lymphoma cells still remain undetermined. In acute myeloid leukemia, loss of miR-199b, a microRNA that targets PCLP1 and discoidin domain receptor 1 and regulates cell migration capacity, has been reported to correlate with poor survival [15].

Mature B-cell non-Hodgkin lymphomas comprise a biologically and clinically heterogeneous group of lymphoid malignancies ranging from indolent to highly aggressive cases, which includes diffuse large B-cell lymphoma (DLBCL), follicular lymphoma (FL), chronic lymphocytic leukemia/small lymphocytic lymphoma (CLL), mantle cell lymphoma (MCL), marginal zone B-cell lymphoma (MZL), Burkitt lymphoma (BL), hairy cell leukemia (HCL), and lymphoplasmacytic lymphoma/Waldenström macroglobulinemia (WM) [16]. The molecular mechanisms that promote mature B cell lymphoma are only partially understood. In the last decades, many genes and signaling pathways whose deregulation induces B cell lymphoma cell proliferation and survival have been elucidated. These include *MYC*, *BCL2* and *BCL6* genes and PI3K, BCR/BTK and the nuclear factor-kB (NF-kB) signaling pathways, among others [17]. Despite progress in the treatment of mature B-cell lymphomas experienced with the addition of anti-CD20 monoclonal antibodies (mAb) to the standard therapy, the prognosis for patients with aggressive forms of the disease still remains poor due to the acquisition of drug resistance [18].

Cancer cells undergo specific alterations in their metabolic pathways to increase the synthesis of proteins, lipids, and nucleic acids necessary to sustain their high proliferation rate [19]. In addition, the metabolic shift allows tumor cells to maintain the redox balance through the generation of reducing molecules, thereby protecting cells from apoptosis [19]. One of the most significant metabolic changes consists in the enhancement of glucose uptake and aerobic glycolysis, referred to as the Warburg effect [19]. Tumor cells also exhibit an upregulation in glutamine import and glutaminolysis for the synthesis of macromolecules [20]. In lymphoma cells, the uptake and metabolism of these nutrients essential for tumor growth depend mainly on MYC, PI3K, and p53 pathway activity [21]. The metabolic reprogramming in tumor cells contributes to drug resistance and can provide new targets for cancer therapy [21].

The aim of this study was to provide insight to the function of PCLP1 in mature B-cell lymphoma cells. Our findings revealed that PCLP1 expression is up-regulated in malignant cells of some mature B-cell lymphoma patients. Overexpression of PCLP1 increases cell proliferation, cell-to-cell adhesion, colony formation and migration in B-cell lymphoma cells. Furthermore, PCLP1 promotes cell resistance to dexamethasone-, hydrogen peroxide- and obinutuzumab-induced cell death. Interestingly, PCLP1 enhances B-cell lymphoma cell dependence on glutamine and pentose phosphate pathway (PPP) and markedly increases cytosolic lipid droplet production. The present work extends our understanding about the molecular mechanisms of mature B-cell lymphomagenesis.

## RESULTS

### Analysis of PCLP1 expression in mature B-cell lymphomas

**Figure 1:**
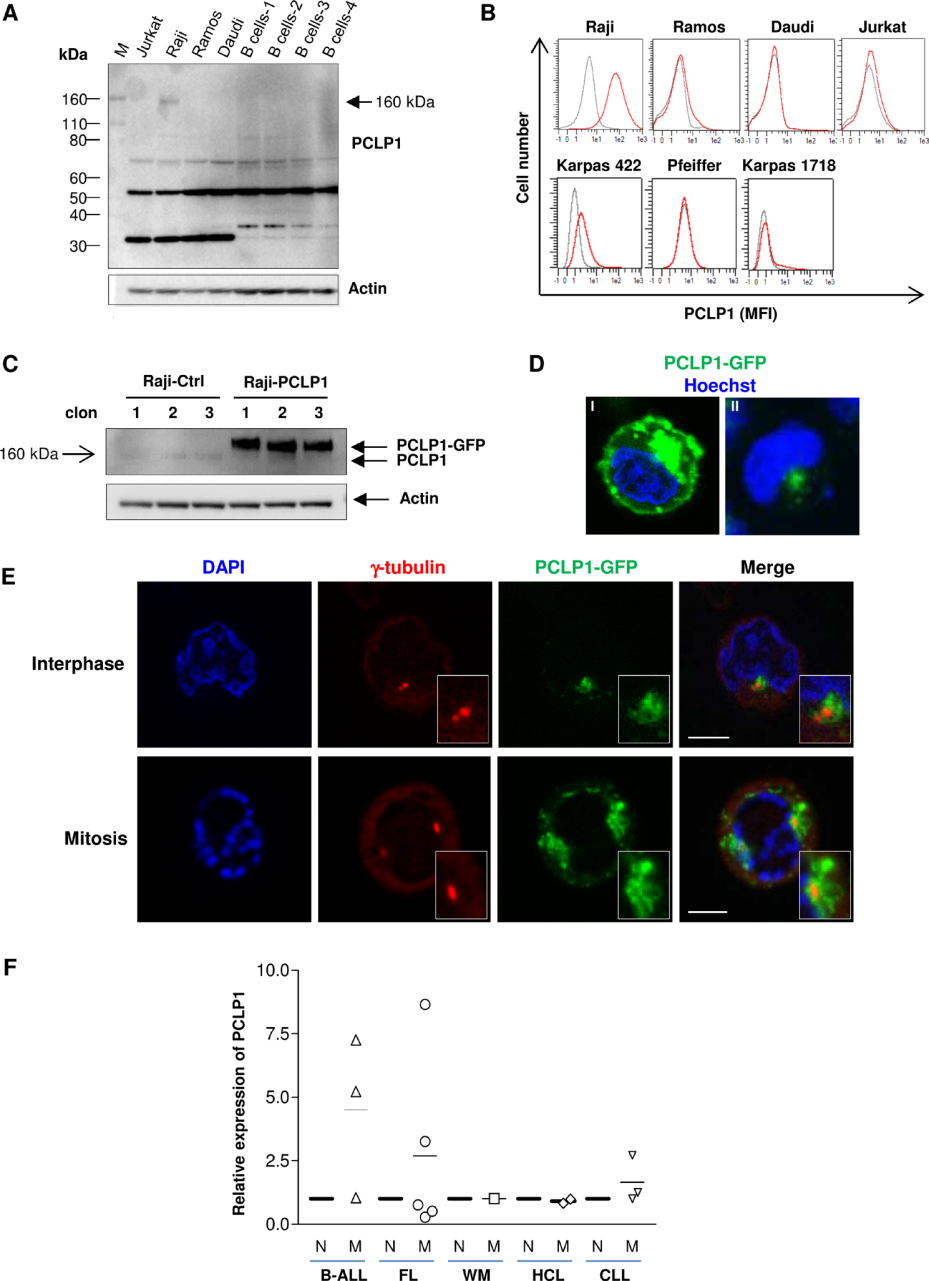
PCLP1 expression in mature B-cell lymphomas (**A**) Western bloting analysis of total PCLP1 expression in B cell lines (Raji, Ramos and Daudi), B cells from four healthy donors (B cells 1–4), and a T cell line (Jurkat). Actin is shown as a loading control. (**B**) PCLP1 expression on the surface of various B cell lines and Jurkat T cell line by flow cytometry. The grey line and the red line in the histograms represent isotype control (mouse IgG) and PCLP1 staining (anti-PCLP1 mAb), respectively. (**C**) Raji cells were stably transfected with pEGFP-PCLP1 (Raji-PCLP1) or pEGFP (Raji-Ctrl) and whole cell extracts from three different clones of each cell type were analyzed for the expression of PCLP1 by Western-blotting using an anti-hPCLP1 mAb. PCLP1: endogenous PCLP1. PCLP1-GFP: ectopic PCLP1. Actin is shown as a loading control. (**D**) Representative fluorescence microscope images of Raji cells stably expressing PCLP1-GFP fusion protein showing the localization of PCLP1-GFP (green) to cell surface and vesicles (I), and centrosome (II), using a 60× objective. Nuclei were stained with Hoechst 33342 (blue). (**E**) Representative fluorescence microscope images of Raji-PCLP1 cells showing the centrosomal localization of PCLP1-GFP fusion protein (green) at interphase (top) and mitosis (bottom). Cells were stained with an anti-γ-tubulin mAb to visualize the pericentriolar area of the centrosome (red). Nuclei were stained with DAPI (blue). Insets at lower right corner show amplified images of the centrosomal region. The size bar indicates 5 µm. (**F**) PCLP1 expression was determined on the surface of malignant and normal B cells from 11 patients with different lymphoma subtypes and 3 patients with ALL by flow cytometry. Median fluorescence intensity (MFI) of PCLP1 expression on malignant B cells was normalized to that detected on normal B cells from the same patient. N: normal B cells; M: malignant B cells; B-ALL: B-cell acute lymphoblastic leukemia; FL: follicular lymphoma; WM: Waldenström macroglobulinemia; HCL: hairy cell leukemia; CLL: chronic lymphocytic leukemia.

We first determined PCLP1 expression in BL lines Raji, Ramos and Daudi, and Jurkat T-lymphoma cell line by Western-blot analysis of total cell lysates. Although the predicted molecular mass of PCLP1 is 55 kDa, the extensive post-translational modification with sialylated oligosaccharides gives rise to a protein with an apparent molecular weight of 160 kDa [22]. The results showed a highly glycosylated form of 160 kDa PCLP1 in Raji cells that was undetectable in the other lymphoma cell lines and normal B cells (Figure 1A). Additional bands of around 70 kDa and 55 kDa were detected in the four lymphoma cell lines examined and in B cells from healthy donors, which may correspond to an intermediate-glycosylated and the unglycosylated forms of PCLP1, respectively. Furthermore, bands of a lower molecular weight than 40 KDa were also observed in all cell types tested, likely representing proteolytic products (Figure 1A). Next, we determined cell surface expression of PCLP1 on the aforementioned cell lines by flow cytometry, extending the analysis to include the diffuse large B-cell lymphoma cell lines Karpas 422 and Pfeiffer and the splenic marginal zone lymphoma cell line Karpas 1718. The results showed high levels of PCLP1 expression on the surface of Raji cells and, to a much lower level, in Karpas 422 cells, whereas it was undetectable on the other cell lines tested (Figure 1B), reflecting the heterogeneity described within several lymphoma subtypes.

In order to investigate the subcellular location of PCLP1 in Raji cells, we transfected cells with an pEGFP1-PCLP1 construct or the empty vector and generated three clones stably expressing PCLP1-GFP fusion protein (Raji-PCLP1) or GFP (Raji-Ctrl), respectively. The level of PCLP1 expression was determined by Western-blot analysis, which shows a highly expressed protein of an apparent molecular weight of approximately 180 kDa corresponding to PCLP1-GFP fusion protein (Figure 1C). Fluorescence microscopy examination revealed location of PCLP1-GFP at the plasma membrane, cytoplasmic vesicles and the perinuclear/centrosomal region (Figure 1D) in Raji-PCLP1 cells. In comparison, control GFP localized diffusively throughout Raji-Ctrl cells (data not shown). The expression of ectopic PCLP1 on cell surface was confirmed by flow cytometry using an anti-PCLP1 mAb (Supplementary Figure 1). We further investigated the centrosomal localization of PCLP1-GFP fusion protein at different stages of the cell cycle. The centrosome is the main microtubule-organizing center in animal cells and plays a critical role in mitosis [23]. It comprises two centrioles, termed the mother and the daughter centrioles, surrounded by pericentriolar material. The centrosome duplicates in interphase, and during mitosis the two resulting centrosomes are separated and migrate to the opposite poles of the mitotic spindle to drive proper chromosome segregation into the two dividing cells. Using γ-tubulin staining as a marker of the pericentriolar area, we observed that PCLP1-GFP localizes to the pericentriolar region and the surrounding area at both interphase and mitosis in Raji-PCLP1 cells (Figure 1E). At interphase, PCLP1-GFP displays an asymmetric distribution, localizing preferentially around one of the centrioles. During mitosis, PCLP1-GFP migrates to opposite poles of the cell in association with the centrosomes (Figure 1E).

PCLP1 expression was further analyzed on the surface of mature normal and malignant B cells from a short cohort of patients with the following diagnosis: FL (5), CLL (3), HCL (2) and WM (1). The flow cytometric gating strategy used to select normal and malignant B cells in each lymphoma type is depicted in Supplementary Figure 2. Given that PCLP1 has been previously reported to be expressed on malignant B cells from B-cell acute lymphoblastic leukemia (B-ALL) patients, we included the expression of this protein in 3 ALL patients as a positive procedure control. As expected, in 2 out of 3 patients with B-ALL, malignant B cells expressed higher levels of PCLP1 than normal B cells from the same patient (Figure 1F).The results revealed that PCLP1 expression levels were 3- to 8-fold higher in malignant B cells compared with normal B cells in 2 out of 5 patients diagnosed with FL and 1 out of 3 patients with CLL (Figure 1F and Supplementary Figure 3). In WM and HCL cases, PCLP1 expression in malignant B cells was similar to normal B cells (Figure 1F).

### PCLP1 induces cell growth, clonogenicity, cell-cell adhesion, and chemotactic migration in mature B-cell lymphoma cells

**Figure 2:**
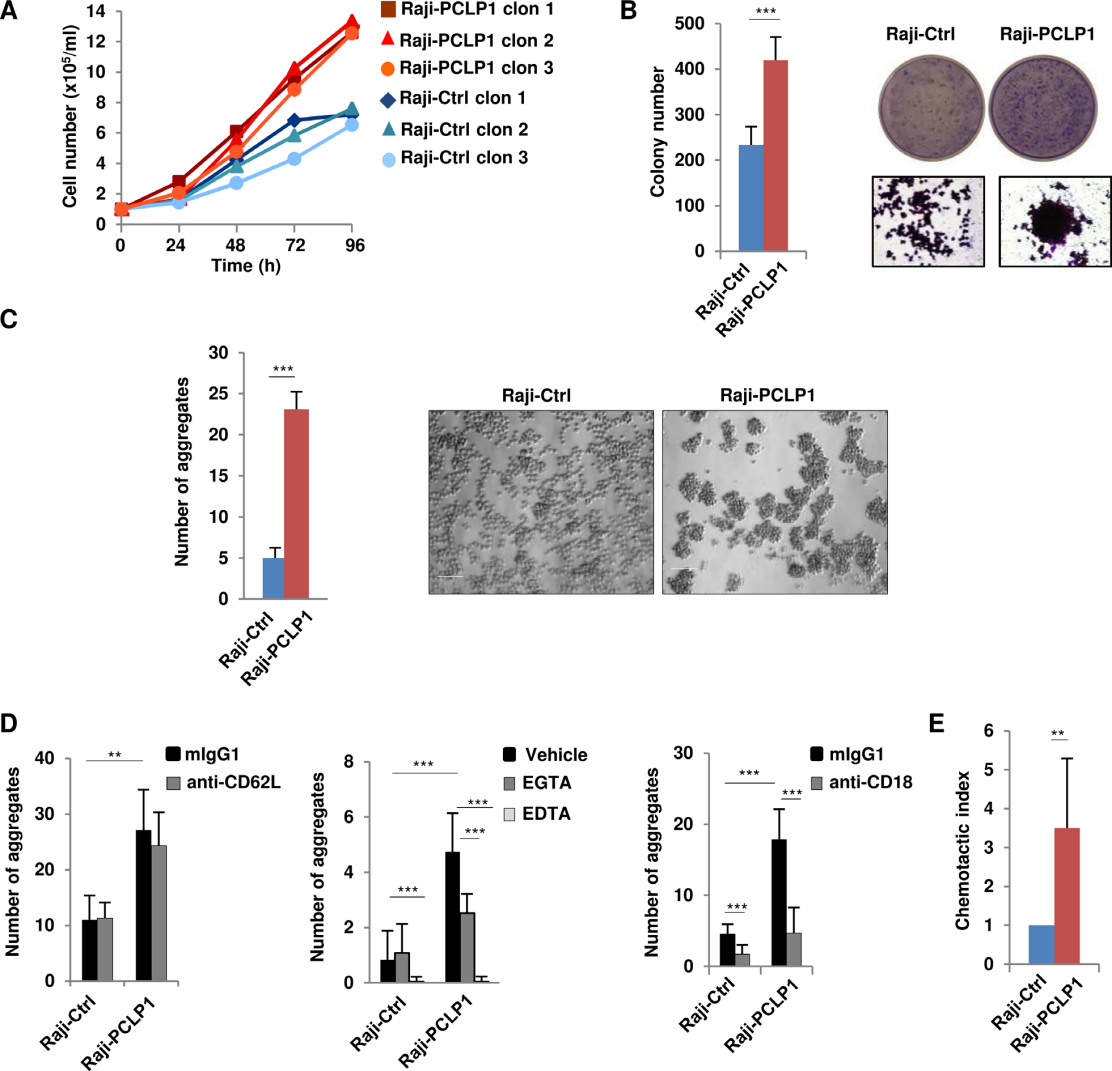
Ectopic expression of PCLP1 induces lymphoma cell proliferation, clonogenicity, cell-to-cell adhesion, and chemotaxis (**A**) Cell proliferation of Raji-PCLP1 and Raji-Ctrl cell clones was measured counting the number of viable cells at different time points of culture in complete medium using the trypan blue exclusion assay. (**B**) Colony formation of Raji-PCLP1 and Raji-Ctrl cells cultured in methylcellulose medium for 11 days. Colonies were stain with crystal violet and counted using an inverted microscope. The graph depicts the number of colonies per well from four independent experiments performed in triplicate. Representative microscope pictures and enlarged images of colony formation after crystal violet staining are shown. Statistical analysis of mean ± SD was calculated using two-tailed paired Student´s *t*-test. ^***^P < 0.001. (**C**) Cell-to-cell adhesion was calculated as the number of cell aggregates formed at 72 h of culture. Representative microscope images of cell aggregates using a 10× objective are shown. The size bar indicates 100 µm. (**D**) Cells were incubated in the presence of anti-CD62L mAb or isotype control (mIgG1) (left) or in the presence of anti-CD18 mAb or mIgG1 (right) and the number of aggregates were counted at 2 h. Cells were cultured in the presence of 1 mM EDTA, 1 mM EGTA or vehicle and the number of aggregates were counted at 24 h (middle). The graphs in C and D represents the mean ± SD of the number of aggregates per field containing more than 20 cells from 3 independent experiment and analyzing 10 fields in each experiment. Statistical analysis was calculated using no-paired Student´s *t*-test (C) and Mann-Whitney *U* test (D). ^**^P < 0.01 ^***^P < 0.001. (**E**) Chemotactic migration of Raji-PCLP1 or Raji-Ctrl toward 100 ng/ml CXCL12 using Transwell chambers. Data represents the mean chemotactic index ± SD normalized to control cells from six independent experiments. Statistical analysis was calculated using Mann-Whitney *U* test. ^**^P < 0.01.

We next aimed at investigating the possible involvement of PCLP1 in mature B-cell lymphomagenesis by analyzing the effect of ectopic PCLP1 expression on cell proliferation and clonogenicity. Raji-PCLP1 cells exhibited increased proliferation (Figure 2A) and higher colony forming ability (Figure 2B) than Raji-Ctrl cells, supporting a role for PCLP1 in B-cell lymphoma cell proliferation and clonogenic growth.

We also observed that Raji cells overexpressing PCLP1 formed numerous large aggregates compared to control cells (Figure 2C), revealing the involvement of PCLP1 in cell-to-cell adhesion in B-cell lymphoma cells. As L-selectin (CD62L) is the only cognate PCLP1 ligand expressed on B cells [4, 24], we performed experiments to evaluate the participation of this molecule in PCLP1-induced cell-to-cell interaction using a function-neutralizing mAb against L-selectin. Treatment of Raji-PCLP1 cells with anti-L-selectin mAb had no effect on PCLP1-induced cell-to-cell adhesion, which pointed to a process independent of L-selectin (Figure 2D, left). We further explored the contribution of integrins to this interaction evaluating the divalent cation-dependency of PCLP1-induced adhesion. The presence of EDTA, a chelator of calcium and magnesium, completely reduced PCLP1-induced adhesion whereas EGTA, a specific chelator for calcium, partially attenuated this adhesion, suggesting an integrin-dependent mechanism (Figure 2D, middle). A blocking antibody against CD18, the β_2_ subunit of leukocyte function-associated antigen-1 (LFA-1) integrin, partially inhibited PCLP1-induced adhesion, indicating the involvement of β_2_-integrin in this process (Figure 2D, right).

Lymphoma cells express the C-X-C chemokine receptor type 4 (CXCR4), which mediates cell migration to organs expressing its ligand C-X-C motif chemokine 12 (CXCL12) [25]. PCLP1 has been reported to associate with CXCR4 and to enhance CXCL12-mediated migration in mouse primary hematopoietic cells [26]. Similarly, we observed that Raji-PCLP1 cells displayed enhanced migration towards CXCL12 compared to Raji-Ctrl cells (Figure 2E). This effect was not due to an increase in CXCR4 expression induced by PCLP1, as Raji-PCLP1 and Raji-Ctrl cells exhibited similar levels of CXCR4 on their surface (Supplementary Figure 4). These results indicate that PCLP1 promotes B-cell lymphoma cell migration towards CXCL12 without altering the expression of CXCR4.

### PCLP1 expression augments mature B-cell lymphoma cell resistance to dexamethasone and reactive oxygen species

**Figure 3:**
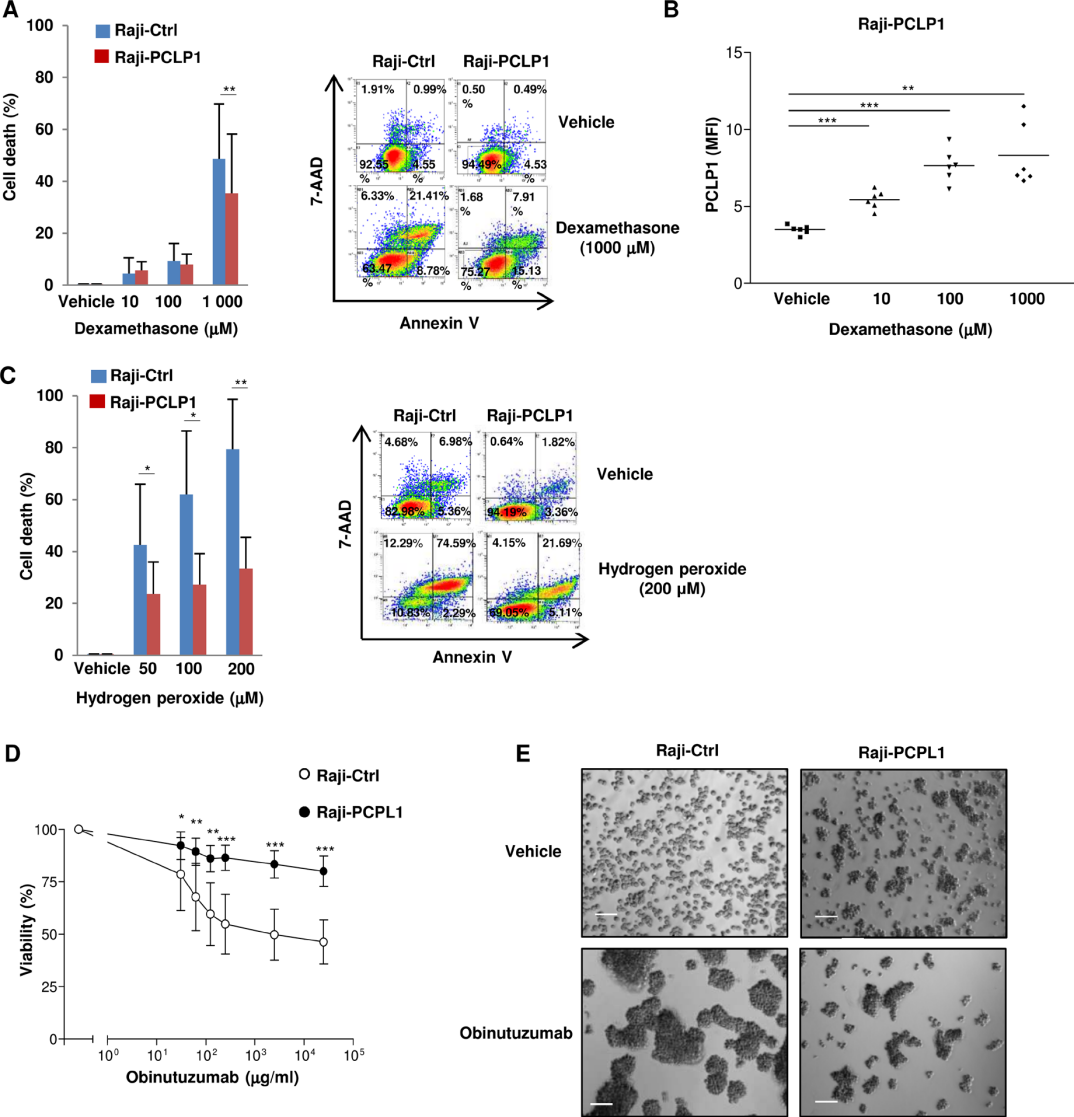
PCLP1 provides resistance to dexamethasone, reactive oxygen species and obinutuzumab-induced death in B-cell lymphoma cells (**A**) Raji-Ctrl and Raji-PCLP1 cells were treated with increasing concentrations of vehicle or dexamethasone for 72 hours and cell death was analyzed after Annexin V-PE/7´AAD staining by flow cytometry. The graph on the left shows the mean percentages ± SD of total specific cell death (including early apoptosis, late apoptosis and necrotic cells) from six independent experiments after subtracting the spontaneous cell death (vehicle). The percentage of specific cell death was calculated as follows: [(% lysis of target cell – % spontaneous cell death)/(100% – % spontaneous cell death)] × 100. Representative dot plots showing Annexin V/7AAD staining in cells treated with vehicle or 1000 µM dexamethasone are shown (right). As displayed in the dot plots, the spontaneous cell death (Annexin V PE positive/7ADD negative, Annexin V PE positive/7ADD positive and Annexin V PE negative/7ADD positive cells) in cells cultured with the vehicle was approximately 7.5% in Raji-Ctrl and 5.5% in Raji-PCLP1 cells. (**B**) Expression of PCLP1 on Annexin V and 7ADD double-negative population of cells treated with dexamethasone or vehicle by flow cytometry. (**C**) Raji-Ctrl and Raji-PCLP1 cells were treated with increasing concentrations of vehicle or H_2_O_2_ for 6 h and cell death was analyzed as indicated in A. The graph on the left shows the mean percentages ± SD of total cell death from five independent experiments after subtracting the spontaneous cell death (vehicle). Representative dot plots showing Annexin V/7AAD staining in cells treated with vehicle or 200 µM H_2_O_2_ are shown (left). As displayed in the dot plots, the spontaneous cell death in the presence of vehicle was approximately 17.0% in Raji-Ctrl and 5.9% in Raji-PCLP1 cells. (**D**) Cells were incubated overnight with increasing concentrations of obinutuzumab or vehicle and cell viability was measured based on Annexin V/7AAD double negative staining relative to vehicle-treated cells. Data show the mean percentages ± SD of viable cells from six independent experiments. (**E**) A representative image of the homotypic adhesion induced by obinutuzumab as visualized by light microscopy is shown. The size bar indicates 100 µm. Statistical analysis was calculated using two-tailed paired Student´s *t*-test. ^*^P < 0.05. ^**^P < 0.01. ^***^P < 0.001.

Glucocorticoids such as dexamethasone have been widely used to treat haematological malignancies including B-cell lymphoma and leukemia, although the development of therapy resistance restricts their effectiveness [27]. Given that PCLP1 has been recently involved in chemoresistance to cisplatin [12, 13], we next aim at determining the participation of PCLP1 in B-cell lymphoma resistance to dexamethasone. Raji cells were subjected to increasing concentrations of dexamethasone for 72 h. Subsequently, dexamethasone-induced cell death was assessed using Annexin V/7AAD staining. The treatment with high concentrations of dexamethasone yielded a reduction of cell death in Raji-PCLP1 cells compared to empty vector-transfected cells (Figure 3A). In accordance with this result, Raji-PCLP1 cells that survive to dexamethasone treatment displayed increased levels of PCLP1 compared to untreated cells, likely due to the death of PCLP1 low-expressing cells (Figure 3B). Altogether, these data indicate that PCLP1 expression confers resistance to high concentrations of dexamethasone in B-cell lymphoma cells.

As glucocorticoids induce lymphoma cell apoptosis through the production of hydrogen peroxide (H_2_O_2_), we then assessed the effect of PCLP1 expression on H_2_O_2_-induced apoptosis [28]. Flow cytometry analysis revealed that apoptotic response to H_2_O_2_-induced oxidative stress was significantly reduced in Raji-PCLP1 cells compared to control cells (Figure 3C). These findings demonstrate that PCLP1 partially inhibits reactive oxygen species-induced apoptosis in B-cell lymphoma cells.

### PCLP1 increases B-cell lymphoma cell resistance to direct programmed cell death induced by obinutuzumab

We next examined the effect of PCLP1 expression on B-cell lymphoma cell susceptibility to anti-CD20 antibody-induced cell death. A recent study demonstrated that obinutuzumab, a novel type II anti-CD20 mAb, induces higher levels of direct, non-complement dependent cell death in various B-lymphoma cell lines and primary B-cell malignancies compared to rituximab [29, 30]. Obinutuzumab-induced cell death is mediated by lysosomes and critically depends on its ability to promote homotypic adhesion in an actin-dependent manner [31]. As PCLP1 is linked to actin cytoskeleton [32], we investigated whether this protein could modulate obinutuzumab-induced cell death. Flow cytometric analysis of cells stained with Annexin V/7AAD showed that Raji-PCLP1 cells displayed a marked increase in the resistance to obinutuzumab-induced cell death compared to Raji-Ctrl cells (Figure 3D). Accordingly, light microscopy examination revealed that Raji-Ctrl cells treated with obinutuzumab experienced a prominent increase in homotypic adhesion, visualized through the presence of large clusters of cells, indicative of an elevated cell death (Figure 3E, left). On the contrary, Raji-PCLP1 cells exhibited a modest increase in homotypic adhesion in response to obinutuzumab treatment (Figure 3E, right). These data demonstrate that PCLP1 expression inhibits obinutuzumab-induced cell death.

### PCLP1 regulates B-cell lymphoma cell metabolism

**Figure 4:**
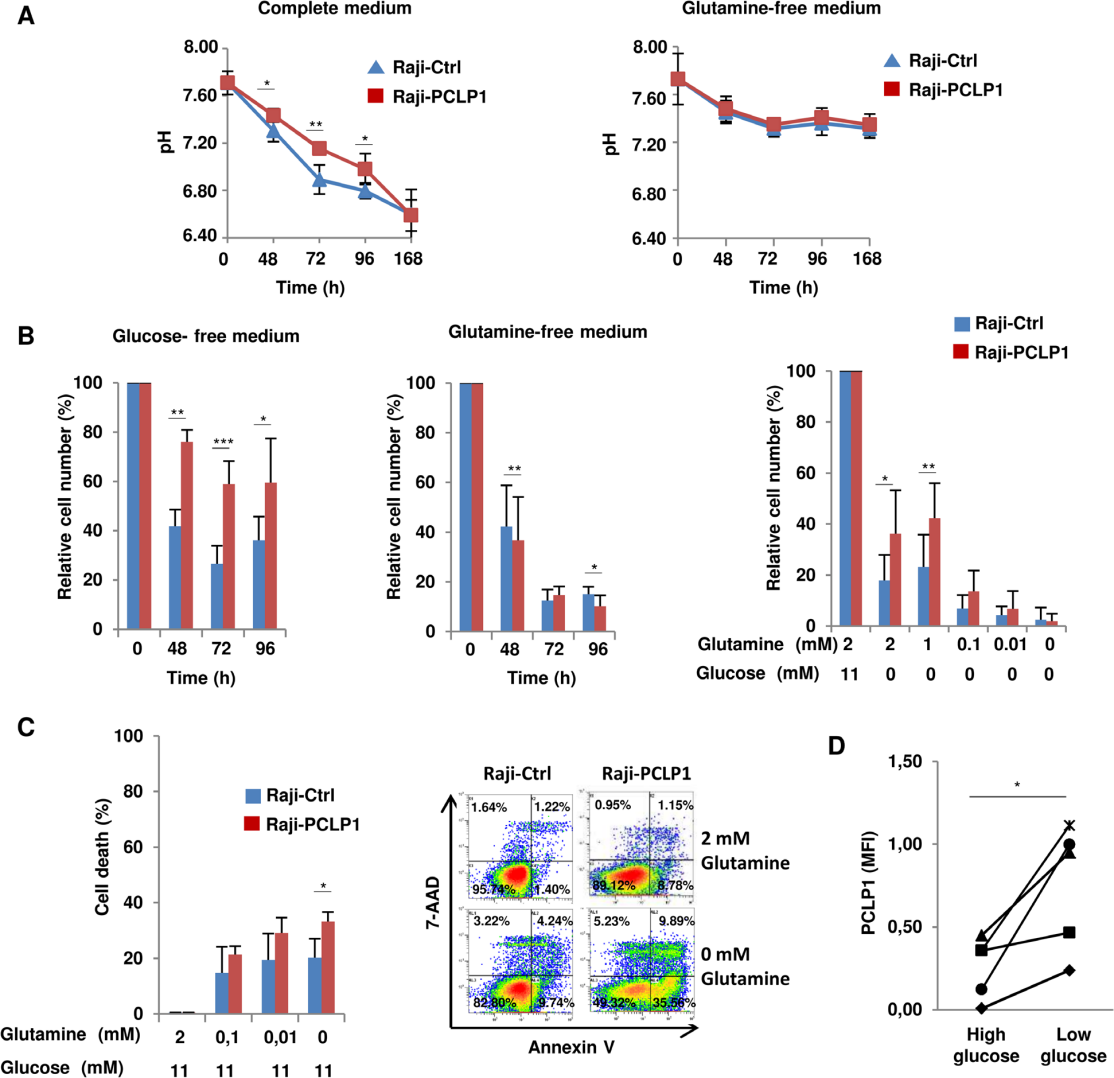
PCLP1 regulates B-cell lymphoma cell metabolism (**A**) Measurement of pH of Raji-PCLP1 or Raji-Ctrl cell culture medium containing glutamine (complete medium) (left) or in the absence of glutamine (right) at the indicated time points. (**B**) Proliferation of Raji-PCLP1 and Raji-Ctrl cells in glucose-free culture medium (left) or glutamine-free culture medium (middle) at different time points. The number of viable cells was normalized to their counterparts grown in complete medium. Data show the mean ± SD of five independent experiments. Proliferation of Raji-PCLP1 and Raji-Ctrl cells in glucose-free conditions and decreasing concentrations of glutamine at 72 h of culture (right). The number of viable cells was normalized to their counterparts grown in complete medium. Data show the mean ± SD of six independent experiments. (**C**) Death of Raji-PCLP1 and Raji-Ctrl cells under glutamine-deprivation conditions. Cells were incubated in the presence of glucose and decreasing concentration of glutamine and the percentage of cell death was determined at 72 h of culture after Annexin V-PE/7´AAD staining by flow cytometry. Data show the mean ± SD of five independent experiments. Representative dot plots showing Annexin V/7AAD staining in cells treated with complete medium (2 mM glutamine/11 mM glucose) or glutamine free medium (0 mM glutamine/11 mM glucose) are depicted. (**D**) Effect of glucose depletion on PCLP1 expression in Raji cells. Cells were cultured in high-glucose medium (11 mM) or low-glucose medium (0.5 mM) for 72 h and the expression of PCLP1 on cell surface was determined by flow cytometry. Data show MFI of PCLP1 expression from five independent experiments. Statistical analysis was calculated using two-tailed paired Student´s *t*-test. ^*^P < 0.05. ^**^P < 0.01. ^***^P < 0.001.

Proliferating tumor cells display increased glucose consumption and lactate production, resulting in acidification of the extracellular medium [19]. However, contrary to expected, we observed a reduction in the acidification of the culture medium from Raji-PCLP1 cells over time compared to Raji-Ctrl cells, as revealed by the retarded change of phenol-red pH indicator to yellow color and confirmed by pH quantification (Figure 4A, left), suggesting that PCLP1 regulates pH homeostasis in B-cell lymphoma cells. Recently, tumor cells have been shown to maintain pH homeostasis by producing ammonia through glutaminolysis [33]. To analyze whether PCLP1 regulates pH homeostasis through this metabolic pathway, Raji-PCLP1 and Raji-Ctrl cells were cultured in medium without glutamine. In the absence of glutamine, no differences in medium pH were found between Raji-PCLP1 cells and Raji-Ctrl cells (Figure 4A, right). These findings point to a role for PCLP1 in regulating tumor cell pH homeostasis through a process dependent on glutaminolysis.

The preceding finding suggests that PCLP1 could activate glutaminolysis pathway and increase the dependency of B-cell lymphoma cells on glutamine to survive and proliferate. PCLP1 activates MAPK oncogenic pathway [10], which is known to regulate glutamine metabolism and cell proliferation. Glutamine represents an alternative source of energy and macromolecular synthesis in tumor cells deprived of glucose [34]. In the absence of glucose, Raji-PCLP1 cells showed a significantly increase in cell proliferation compared to Raji-Ctrl cells (Figure 4B, left). On the contrary, in glutamine-deprivation conditions, the proliferative capacity of Raji-PCLP1 cells decreased slightly but significantly compared to that of Raji-Ctrl cells (Figure 4B, middle). When cells were cultured under glucose-free conditions with decreasing concentrations of glutamine, the enhanced effect of PCLP1 on cell proliferation gradually diminishes to the levels observed in Raji-Ctrl cells, indicating that the increase in cell proliferation induced by PCLP1 in the absence of glucose is dependent on glutamine (Figure 4B, right). We next evaluated the effect of PCLP1 expression on Raji cell survival in glutamine-deprivation conditions. Decreasing concentrations of glutamine in the presence of glucose yielded an increase in total cell death in Raji-PCLP1 cells compared to Raji-Ctrl cells (Figure 4C). Altogether, these results demonstrate that PCLP1 induces glutamine dependency and reduces glucose dependency in B-cell lymphoma cells.

In human podocytes, high-glucose levels have been shown to dramatically down-regulate PCLP1 expression which is restored to normal values when cultured under low-glucose conditions [35, 36]. We therefore explored the effect of glucose depletion on PCLP1 expression in B-cell lymphoma cells. Flow cytometry analysis showed that Raji cells experienced an increase in surface PCLP1 expression when grown in low-glucose medium (0.5 mM) compared to high-glucose medium (11 mM) (Figure 4D). These data indicate that glucose deprivation enhances PCLP1 surface expression in B-cell lymphoma cells.

### PCLP1 expression induces lipid droplet synthesis

**Figure 5:**
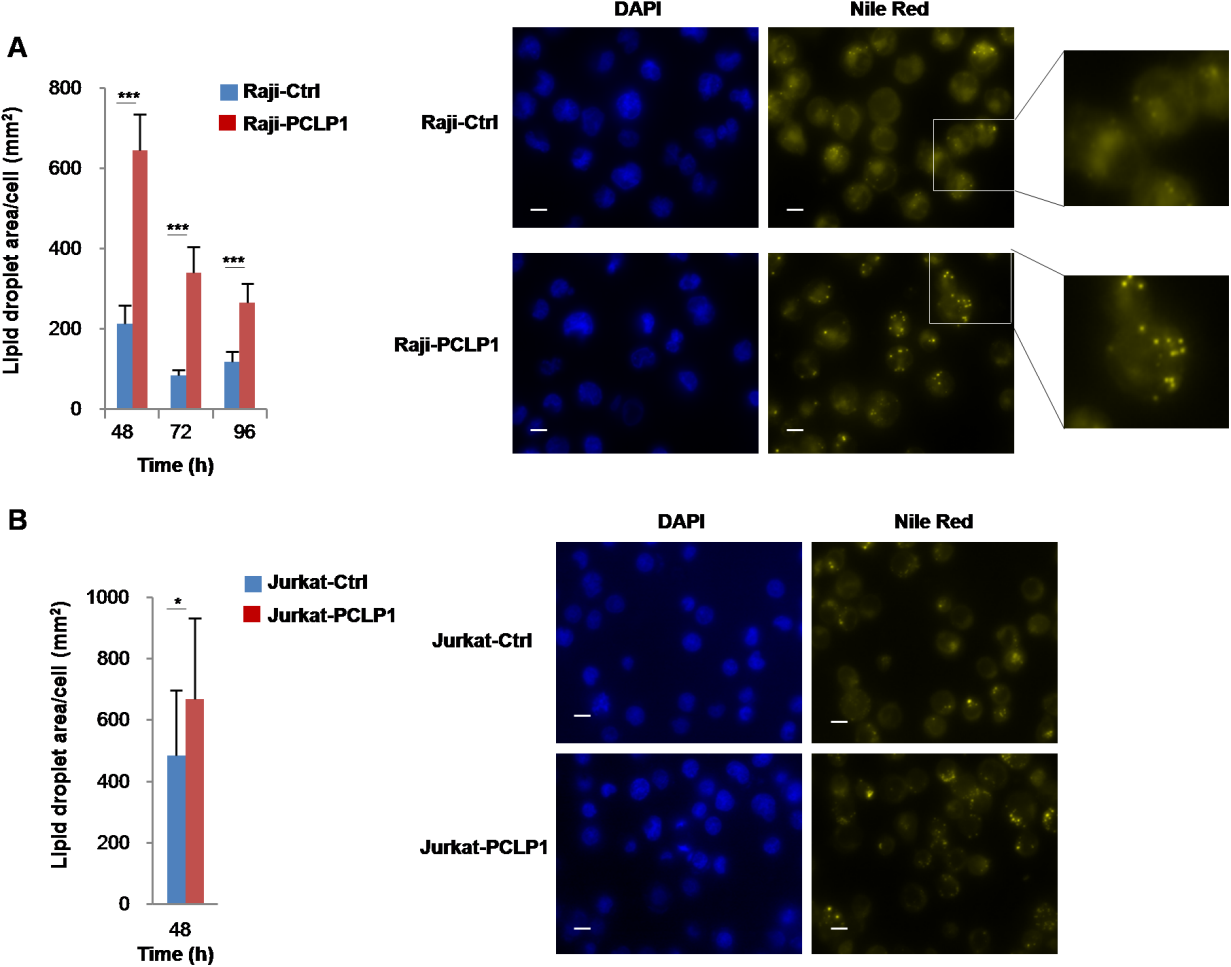
PCLP1 induces lipid droplet synthesis Determination of lipid droplet content in Raji-PCLP1 and Raji-Ctrl cells (**A**) or Jurkat-PCLP1 and Jurkat-Ctrl cells (**B**) by fluorescence microscopy. Cells were cultured in complete medium for the indicated times, fixed and stained with Nile Red for the detection of neutral lipid droplets. Ten fields were randomly selected using a 60× oil objective and the total area of lipid droplets per cell was calculated employing ImageJ software. Bar graphs represent the quantitative analysis of lipid droplets content per cell. Mean ± SD of lipid droplet area per cell is shown. Statistical analysis was calculated using Mann-Whitney *U* test. ^*^P < 0.05. ^**^P < 0.01. ^***^P < 0.001. Representative fluorescence microscopy images depicting lipid droplets stained with Nile Red (right panels, yellow). Nuclei were labeled with DAPI (left panels, blue). Digital zoom of a selected area is shown. The size bar indicates 10 µm.

Alteration of lipid metabolism is a hallmark of many tumors. Highly proliferative tumor cells enhance the endogenous synthesis of lipids, which excess store in the form of lipid droplets [37]. The examination of Raji-PCLP1 cells by bright-field microscopy revealed the presence of a higher number of cytoplasmic vesicles compared to Raji-Ctrl cells (data not shown). To unveil the vesicle composition, cells were stained with the lipophilic dye Nile Red, a marker of neutral lipids, at different time points of culture in complete medium. Fluorescence microscopy analysis showed a markedly increased accumulation of cytoplasmic neutral lipid droplets in Raji-PCLP1 cells at 48 h of culture as compared to Raji-Ctrl cells (Figure 5A). Afterwards, lipid stores gradually decreased over time, probably due to lipolysis and lipid oxidation induced by nutrient starvation. The overexpression of PCLP1 also increased lipid storage in Jurkat human acute leukemic T cells (Figure 5B), albeit to a lower extent than in Raji cells, which indicates that PCLP1-induced lipid droplet formation is not exclusive of B-cell lymphomas.

### PCLP1-induced cell proliferation is decreased by metabolic inhibitors

**Figure 6:**
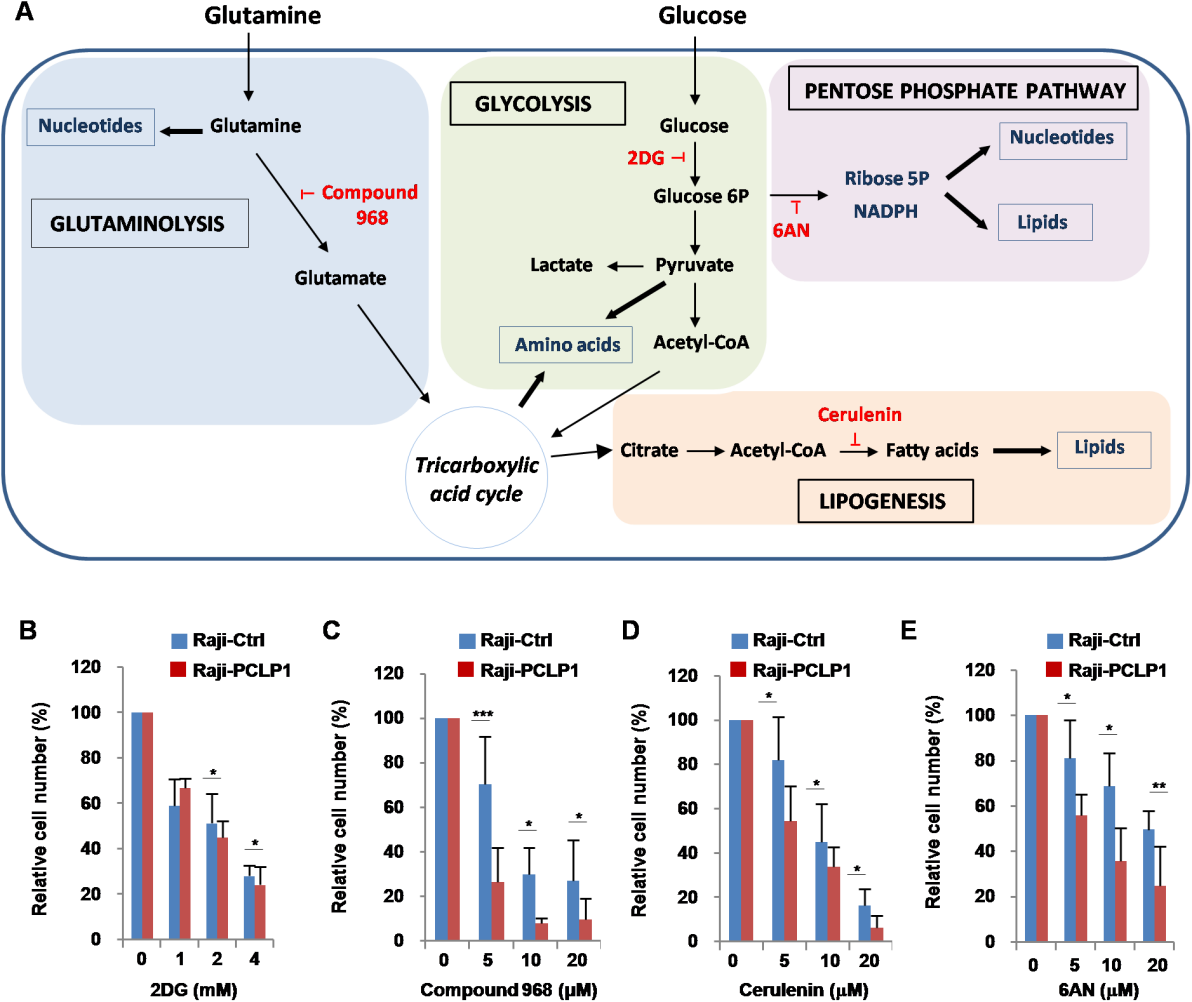
Effect of metabolic inhibitors on PCLP1-induced cell proliferation (**A**) Schematic representation of cancer cell metabolism. Once transported into cells, glucose is phosphorylated to glucose 6-phosphate by hexokinases. Glucose 6-phosphate can then be metabolized via aerobic glycolysis or PPP. Through the aerobic glycolysis, glucose 6-phosphate is sequentially converted to pyruvate. Afterwards, pyruvate is transformed to acetyl-CoA via TCA or to lactate. Acetyl-CoA can be used for the synthesis of fatty acids and lipids for biomembrane generation. Glucose 6-phosphate that enters PPP generates ribose, for nucleotides synthesis, and NADPH, for FA synthesis. Glutamine provides intermediates to maintain TCA function and contributes to lipid synthesis and the production of purines and pyrimidines for nucleotide synthesis. Glucose and glutamine also provide the carbon and nitrogen necessary for the synthesis of nonessential amino acids. Metabolic inhibitors are depicted in red color. 2DG: 2-deoxyglucose; 6AN: 6 aminonicotinamide. (**B**) Raji-PCLP1 and Raji-Ctrl cells were cultured for 72 h in complete medium in the absence or in the presence of increasing concentrations of the 2-DG (glycolysis inhibitor). (**C**) To determine the effect of compound 968 (glutaminase inhibitor) on cell proliferation, Raji-PCLP1 and Raji-Ctrl cells were cultured for 7 days in complete medium in the absence or in the presence of increasing concentrations of the inhibitor. (**D**) Raji-PCLP1 and Raji-Ctrl cell were cultured for 72 h in complete medium in the absence or in the presence of increasing concentrations of cerulenin (fatty acid synthase inhibitor). (**E**) Raji-PCLP1 and Raji-Ctrl cells were cultured for 72 h in complete medium in the absence or in the presence increasing concentrations of 6AN (PPP inhibitor). Cell proliferation was assessed by counting live cells on a hemocytometer using trypan blue exclusion dye. The number of viable cells was normalized to their counterparts grown in the absence of the inhibitor. Data show the mean ± SD percentage relative to non-treated cells of five independent experiments. Statistical analysis was calculated using two-tailed paired Student´s *t*-test. ^*^P < 0.05. ^**^P < 0.01. ^***^P < 0.001.

We next sought to examine the metabolic pathways involved in PCLP1-induced B-cell lymphoma cell proliferation using specific inhibitors that target key metabolic enzymes (Figure 6A). Therefore, Raji-PCLP1 cells and Raji-Ctrl cells were treated with increasing doses of 2-deoxy-D-glucose (2DG), a well-characterized glycolysis inhibitor. Cell proliferation was slightly decreased in Raji-PCLP1 relative to Raji-Ctrl cells in response to 2DG (Figure 6B). To evaluate the dependence of PCLP-induced B-cell lymphoma cell proliferation on glutaminolysis, we tested its sensitivity to Compound 968, a selective inhibitor of glutaminase 1 (GLS1). GLS1, the first enzyme in glutaminolysis pathway, converts glutamine to glutamate and plays a crucial role in cell proliferation. Treatment with Compound 968 decreased cell proliferation in Raji-PCLP1 cells to a significantly greater extent than in Raji-Ctrl cells (Figure 6C). These results indicate that glutaminolysis is essential for the increase of B-cell lymphoma cell proliferation induced by PCLP1.

Contrary to normal cells, which prefer exogenous fatty acids, tumor cells depends on *de novo* fatty acid synthesis from tricarboxylic acid cycle-derived precursors catalyzed by fatty acid synthase (FASN) [38]. Fatty acids are used for the synthesis of membranes and signaling molecules, oxidized to produce energy, or stored in lipids droplets. As shown in Figure 6D, Raji-PCLP1 cells treated with cerulenin, a FASN inhibitor, experienced a suppression in proliferation compared to Raji-Ctrl cells, suggesting that PCLP1-induced B-cell lymphoma cell proliferation relies on endogenous fatty acid synthesis.

PPP is an alternative metabolic pathway for glucose metabolism that promotes cancer progression by providing tumor cells with both ribose-5-phosphate, necessary for nucleotide synthesis, and NADPH, a molecule crucial for redox homeostasis maintenance and reductive biosynthesis of lipids and nucleotides [39]. Many cancer cells increase the PPP flux via activation of glucose 6-phosphate dehydrogenase (G6PD), the enzyme catalyzing the first reaction of this pathway [39]. To determine whether PCLP1 induces B-cell lymphoma cell proliferation through PPP, we treated Raji-PCLP1 cells and Raji-Ctrl cells with 6-aminonicotinamide (6AN), a competitive inhibitor of G6PD. The inhibitory effect of 6AN on cell proliferation was more pronounced in Raji-PCLP1 cells than in Raji-Ctrl cells (Figure 6E). Microscopic examination revealed extensive cytoplasmic vacuolation in Raji-PCLP1 cells upon 6AN treatment, which were absent in Raji-Ctrl cells (Supplementary Figure 5, middle panels). The subsequent addition of fresh medium without 6AN to vacuolated Raji-PCLP1 cells led to complete disappearance of the cytoplasmic vacuoles, reflecting the reversibility of the effect (Supplementary Figure 5, bottom panels). These data indicate that PCLP1 enhances the dependency of B-cell lymphoma cell proliferation on PPP.

## DISCUSSION

In this work, we show for the first time that PCLP1 enhances the proliferative and clonogenic potential of mature B-cell lymphoma cells. PCLP1 function revealed in our study resembles that described for other cell-membrane molecules expressed on B-cell lymphomas, such as B7H6, which induces cell proliferation, colony formation, migration and dexamethasone resistance [40]. Our results are in line with previous reports showing that PCLP1 induces cell proliferation in breast cancer, oral squamous cell carcinoma and glioblastoma [41–43]. In breast cancer cells, PCLP1 activates PI3K and MAPK signaling pathways [10], both implicated in proliferation, survival and progression of B-cell non-Hodgkin lymphomas. A recent work using a mouse model has demonstrated the critical need of PI3K pathway activation besides MYC deregulation for the malignant transformation of normal B cells in BL, an aggressive B-cell lymphoma with an extremely high proliferation index [44]. Ras/MAPK pathway also contributes to MYC-induced lymphomagenesis [45]. These observations suggest that PCLP1 might cooperate with MYC to promote lymphomagenesis through the activation of PI3K or/and MAPK pathways in B-cell lymphoma.

We found that PCLP1 induces cell-to-cell adhesion of B-cell lymphoma cells through a process dependent on β_2_-integrin, which correlates with a report demonstrating the dependency of PCLP1-induced adhesion on integrin function [46]. Similarly, a previous study on B-CLL revealed the participation of β_2_-integrin in cell-to-cell adhesion induced by CD19 [47], a major B-cell receptor independent activator of MYC-driven B-cell neoplastic growth [48]. In B-cell lines and normal B cells, adhesion mediated by the interaction of the β_2_-integrin LFA-1 with its ligand intercellular adhesion molecule-1 (ICAM-1) stimulates proliferation and inhibits apoptotic cell death [49]. In the same way, β_2_-integrin-mediated intracellular signals delivered as a consequence of PCLP1-induced cell-to-cell adhesion could promote B-cell lymphoma cell proliferation and survival. Although PCLP1 expressed on high endothelial venules interacts with L-selectin during lymphocyte recruitment [4], the failure of a blocking anti-L-selectin antibody to abrogate PCLP1-induced cell-to-cell adhesion in B-cell lymphoma point to another ligand as responsible for this interaction. Our data also demonstrate that forced expression of PCLP1 enhances B-cell lymphoma cell migration toward CXCL12, suggesting that it might direct the dissemination of lymphoma cells to organs expressing this chemokine, including lymph nodes, lungs, liver and bones.

PCLP1-induced resistance to dexamethasone could be due to both the direct activation of PI3K/Akt or MAPK pathways and the generation of survival signals elicited by PCLP1-induced cell-to-cell adhesion. PI3K/Akt and MAPK signaling pathways have been demonstrated to mediate glucocorticoid resistance in a variety of tumors, including leukemia [50]. In support of our results, a recent report demonstrates that PCLP1 confers osteosarcoma chemoresistance to cisplatin via a PI3K-dependent mechanism [13]. In oral tongue squamous cell carcinoma, PCLP1 induces chemoresistance to cisplatin through a mechanism involving B-cell-specific lymphoma Moloney murine leukemia virus integration site 1 homolog (BMI-1) [12], an oncogene that collaborates with MYC in the development of murine lymphoma [51] and which causes drug resistance in B-cell lymphoma cells [52]. Our study also shows that PCLP1 diminishes obinutuzumab-induced cell death, which has been recently proven to be effective in the treatment of patients with rituximab-refractory indolent non-Hodgkin lymphoma [53]. These observations suggest that strategies aimed at targeting PCLP1 might be beneficial for the treatment of dexamethasone- and obinutuzumab-resistant B-cell lymphomas expressing PCLP1.

To our knowledge, this is the first study reporting a role for PCLP1 in cancer metabolism reprogramming. We demonstrate that PCLP1 induces glutamine addiction and enhances cell proliferation under glucose-deprived conditions in Raji B-cell lymphoma cells, which correlates with a previous study demonstrating a key role of glutamine in BL lymphoma cell survival and proliferation under glucose deficiency [34]. Glutamine, the most abundant amino acid in human body, is necessary as a nitrogen donor for the synthesis of purines and pyrimidines to form the nucleotides as well as non-essential amino acids required for cell proliferation [20]. PCLP1 may enhance glutamine dependency through the activation of MAPK, known to be involved in glutaminolysis. PI3K as well as MYC act as major determinants of glucose and glutamine metabolism in lymphoma cells by regulating essential enzymes of these metabolic pathways [21]. Furthermore, PI3K/Akt pathway promotes cell survival and proliferation in glucose limiting conditions due to high nutrient consumption and dysfunctional vasculature [54]. PCLP1 might regulate cell response to glucose-starvation conditions through the enhancement of glutaminolysis pathway to support lymphoma cell survival and proliferation. In accordance with this notion, our results show an increase in PCLP1 expression under low-glucose conditions in lymphoma cells.

We observed that PCLP1 increases lipid droplet formation in Raji cells, suggesting the participation of this protein in the accumulation of the cytosolic lipid vacuoles that morphologically characterized BL cells. Our results also demonstrate that B-cell lymphoma cells overexpressing PCLP1 rely on FASN activity to proliferate, which correlates with a previous study reporting up-regulated expression and activity of FASN in these tumor cells [55]. Enhanced FASN expression has been associated with tumor metastasis, chemoresistance and decreased patient survival in many tumors [56]. Under adverse environmental conditions, such as hypoxia, tumors cells increase the uptake of glutamine to synthesize *de novo* high amounts of lipids that are accumulated in lipid droplets to protect cells from oxidative and endoplasmic reticulum stress [57]. Following re-oxygenation, tumor cells uses the stored lipids to support cancer cell proliferation [57]. These observations suggest that PCLP1 could induce chemoresistance through the deregulation of lipid metabolism.

Our study shows that PCLP1 overexpressing cells depend on PPP to survive and proliferate, suggesting that PCLP1 increases PPP flux. Many tumors, including DLBCL, display an enhanced PPP flux and overexpression of G6PD [58]. PPP up-regulation enhances tumor cell survival, proliferation, metastasis and chemoresistance and is associated with aggressive forms of cancers [39]. PPP provides cells with ribose-5-phosphate for the synthesis of nucleotides required to sustain rapid cell proliferation, and NADPH for reactive oxygen species neutralization and biosynthesis of lipids and deoxyriboses [39]. Thus, the shift to PPP flux in PCLP1 expressing lymphoma cells could explain the increase cell proliferation, lipid synthesis and resistance to dexamethasone observed in our study.

We also show that PCLP1 localizes to the pericentriolar material region, the centrosome component necessary for microtubule assembly and spindle formation. PCLP1 interacts with both ezrin [32], which crosslink plasma membrane with actin cytoskeleton and is essential for centrosome positioning, and cortactin [59], an adapter protein that mediates actin-driven centrosome separation at cell cycle G2-M transition. Furthermore, PCLP1 activates the small GTPase Cdc42 [60], involved in the modulation of actin and microtubules dynamics, centrosome position, spindle orientation, and chromosome segregation [61]. It is tempting to speculate that PCLP1 could regulate centrosome dynamics, influencing spindle apparatus orientation and, consequently, cell division.

In summary, our study identifies a role for PCLP1 in mature B-cell lymphomagenesis and suggests PCLP1 and the metabolic pathways essential for PCLP1-induced cell survival and proliferation as potential therapeutic targets to improve patient´s survival.

## MATERIALS AND METHODS

### Cell lines and primary cells

Human samples were obtained from patients and healthy donors after informed consent as approved by the Ethical Committee of the hospital and in accordance with the ethical guidelines of the Declaration of Helsinki. Malignant and peripheral blood mononuclear cells (PBMC) were isolated by Ficoll-Hypaque gradient centrifugation. Peripheral B cells were isolated from PBMCs by negative selection using the RosetteSep human B cell enrichment cocktail technique following the manufacture´s instruction (StemCell Technologies, Vancouver, Canada). The purity of isolated B cells was more than 90% as determined by flow cytometry on the basis of CD19 expression and CD3 negativity.

Human Burkitt lymphoma cell lines Raji, Ramos and Daudi, human acute T cell leukemia cell line Jurkat, and human diffuse large B-cell lymphoma cell line Pfeiffer were obtained from American Type Culture Collection (ATCC) (Manassas, Virginia, USA). Human diffuse large B-cell lymphoma cell line Karpas 422 and splenic marginal zone lymphoma cell line 1817 were obtained from Sigma-Aldrich (Sant Louis, Missouri, USA). Raji, Ramos, Daudi, Jurkat, Karpas 422 and Karpas 1718 cells were cultured in RPMI 1640 medium (Lonza, Basel, Switzerland) containing 2 mM glutamine and 11 mM glucose and supplemented with 10% heat-inactivated fetal bovine serum (FBS) (Lonza), 100 U/ml penicillin, and 100 U/ml streptomycin (Sigma-Aldrich) under 5% CO_2_ at 37°C. Pfeiffer cells were cultured in RPMI 1640 medium containing 2 mM glutamine and 11 mM glucose and supplemented with 20% heat-inactivated FBS, 100 U/ml penicillin, and 100 U/ml streptomycin under 5% CO_2_ at 37°C*.*

The World Health Organization (WHO) classification of neoplastic diseases of the hematopoietic and lymphoid tissues was used to classify the tumors, which included FL, WM, ALL, HCL and CLL.

### Cell surface staining

Cells were resuspended in staining solution (PBS supplemented with 0.1% bovine serum albumin (BSA) and 0.01% sodium azide) and incubated with 20 μg/ml human IgG for 15 min at room temperature to block nonspecific binding due to Fc receptors*.* Afterwards, cells were incubated with specific monoclonal antibodies for 30 min at room temperature. For the detection of PCLP1 on Raji cell line in high and low glucose conditions, cells were incubated with a phycoerythrin (PE)-conjugated anti-human PCLP1 mAb (R&D Systems, Minneapolis, Minnesota, USA). For the detection of PCLP1 on B cell lines, cells were incubated with a primary anti-human PCLP1 mAb (R&D Systems) followed by a PE-conjugated anti-mouse IgG polyclonal antibody (R&D Systems). For the detection of PCLP1 on primary patient´s samples, cells were incubated with a primary anti-human PCLP1 mAb (R&D Systems) followed by a PE-conjugated anti-mouse IgG polyclonal antibody (R&D Systems) and the following antibodies: CD45, CD19, CD20 (all samples), CD10 (FL), CD34 and CD10 (LLA), CD103 (HCL), and CD5 (LLC) (BD Biosciences, San José, California, USA). Isotype-matched control antibodies were used to evaluate nonspecific binding. Finally, after fixing with 1% formaldehyde, cells were washed, resuspended in 500 μl PBS, and analyzed on a Cytomics FC500 flow cytometer equipped with the CXP analysis software (Beckman Coulter, Brea, California, USA) or a MACSQuant Analyzer 10 flow cytometer equipped with the MACSQuantify software (version 2.6) (Miltenyi Biotec, Bergisch Gladbach, Germany). CXCR4 was detected using an allophycocyanin (APC)-conjugated anti-human CXCR4 mAb (Biolegend, San Diego, California, USA). A minimum of 5.000 (cell lines) or 30.000 (primary cells) events per sample were acquired. Data are presented as median fluorescence intensity (MFI) corrected for nonspecific staining using fluorescence minus one (FMO) and isotype controls. Gating strategies for flow cytometric immunophenotyping are depicted in Supplementary Figure 2.

### Generation of stable transfected cells

Raji and Jurkat cells were transfected with pEGFP-N1 expression vector encoding hPCLP1 (a gift from Dr. R. Parrilla, CIB-CSIC) or pEGFP-N1 empty vector as a negative control using Lipofectamine^®^ 2000 (Thermo Fisher Scientific, Waltham, Massachusetts, USA), according to the manufacturer´s protocol. Briefly, 1 μg DNA was pre-incubated with 1 μl PlusReagent^®^ (Thermo Fisher Scientific) in 50 μl OptiMEM (Thermo Fisher Scientific) for 10 minutes. Separately, 2 μl Lipofectamine^®^ 2000 was incubated with 50 μl OptiMEM for 5 minutes. Afterwards, both solutions were mixed and added to 1 × 10^5^ cells in 500 ul of RPMI 1640 supplemented with 10% FBS and 1% of non-essential amino acid (NEAA) solution (Gibco®, Thermo Fisher Scientific). Cells were then seeded in 24-well culture plates containing 1 mL of complete RPMI medium and geneticin at a final concentration of 800 μg/ml to select cells stably expressing the indicated vector. At 72 h post-transfection, cells were cultured under limiting dilution conditions in 96-well culture plates to isolate single clones expressing PCLP1. Protein expression was confirmed by flow cytometry, microscopy, and Western blotting. Cells stably expressing pEGFP-PCLP1 or empty vector were routinely grown in complete medium supplemented with geneticin at a final concentration of 400 μg/ml. The maintenance of GFP expression over time was verified by flow cytometry.

### Cell proliferation and clonogenic assay

To measure cell proliferation, cells were seeded at 10^5^ cells /ml in complete, glutamine-free or glucose-free RPMI 1640 medium (Lonza). Then, cells were counted at different time points using a hemocytometer by Trypan-Blue exclusion assay. When indicated, cells were incubated in the presence of the indicated metabolic pathway inhibitors.

Clonogenic assays were performed in triplicate by adding 2 × 10^3^ cells to 3 ml semi-solid medium (RPMI 1640 medium containing 1.5% methylcellulose, 30% FBS, 100 U/ml penicillin, and 100 U/ml streptomycin). The mixture was then plated in a 6-well plate and maintained in culture at 37°C with 5% CO2 for 11 days to allow colony formation. Colonies consisting of more than 50 cells in each well were counted using an inverted microscope. Images were captured after fixing the cells with 95% methanol and staining with 0.5% crystal violet.

### Cytotoxic assays

Cells were incubated with different concentrations of dexamethasone (Sigma-Aldrich), hydrogen peroxide (Industrias Noriega, Asturias, Spain), obinutuzumab (a gift from Roche), 6-Aminonicotinamide (6AN) (Vitro, Madrid, Spain) or under conditions of decreasing concentrations of glutamine (Thermo Fisher Scientific) for different time points. Cell death was determined by staining with PE-conjugated Annexin V and 7-amino actinomycin D (7AAD) (Immunostep, Salamanca, Spain) and analyzed by flow cytometry. The percentage of cell death, including early apoptosis, late apoptosis and necrosis (Annexin V PE positive/7ADD negative, Annexin V PE positive/7ADD positive and Annexin V PE negative/7ADD positive cells) was calculated after subtracting the percentage of spontaneous death of cells incubated with the vehicle from the total cell death using the following formula: % specific lysis = [(% lysis of target cell - % spontaneous cell death)/(100% – % spontaneous cell death)] × 100.

### Cell-to-cell adhesion

Disaggregated cells were seeded at a density of 1 × 10^5^ cells/ml in 8 mL of complete RPMI 1640 medium and the formation of aggregates was examined at 72 hours of culture by phase contrast with a 10 × objective. To examine the effect of divalent cations on aggregate formation, cells were cultured in the presence or absence of 1 mM EDTA (Sigma-Aldrich) or EGTA (Sigma-Aldrich) for 24 h. For antibody-blocking experiments, cells were placed in 96-well culture plates at a density of 0.5–1 × 10^6^ cells/ml in 100–150 μl/well of complete RPMI 1640 medium containing 10 µg/ml anti-CD18 (Biolegend), anti-CD62L (BioLegend) mAbs or mouse IgG isotype control (BioLegend) and then incubated at 37°C for 2 h. Photomicrographs were taken using an ORCA-ER monochrome cooled-CCD camera (Hamamatsu, Hamamatsu, Japan) coupled to an Eclipse TE2000-E inverted fluorescence microscope (Nikon, Tokyo, Japan) and NIS-Elements AR imaging software and the number of aggregates containing more than 20 cells per field was counted.

### Chemotaxis assay

Cell chemotaxis was monitored using 6.5-mm-diameter 24-Transwell chemotaxis chambers with polycarbonate filters of 5-μm pore size (Corning, Corning, New York, USA). Briefly, the bottom compartment was filled with 600 μL of assay buffer (serum-free RPMI-1640 medium with 1% BSA) containing 100 ng/ml of CXCL12, and 5 × 10^5^ tumor cells in 100 μL of assay buffer were seeded in the upper compartment. A negative control without CXCL12 was included to assess random migration*.* After 3 h at 37°C in a 5% CO2 atmosphere, the cells that had migrated to the bottom compartment were collected and counted by flow cytometry. The chemotaxis index was calculated by dividing the number of migrating cells in the presence of CXCL12 by the number of migrating cells in the absence of chemoattractant.

### Western blotting

Cells were washed with cold PBS and lysed in ice-cold Igepal lysis buffer (1% Igepal, 20 mM TrisHCL, 140 mM NaCl, 1 mM EDTA, pH 7.4) containing complete protease inhibitor cocktail (Sigma) and 1mM phenylmethanesulfonyl fluoride (PMSF) for 30 min on ice. After centrifugation at 13,000 g for 20 min at 4°C, the supernatant was collected and protein concentration determined by Bradford Protein Assay (Bio-Rad, Hercules, California, USA) using a POLARstar Omega microplate reader (BMG Labtech, Ortenberg, Germany). Equal amounts of total protein were subjected to 4%–12% sodium dodecyl sulphate polyacrylamide gel electrophoresis (SDS-PAGE) and transferred to a polyvinylidene difluoride membrane using the iBlot Dry Blotting System (Invitrogen, Carlsbad, CA, USA). Next, the membranes were blocked with 1% BSA in Tris-buffered saline Tween 20 and incubated overnight with an anti-podocalyxin mAb (Santa Cruz Biotechnology, Dallas, Texas, USA) diluted 1:200 in blocking solution at 4°C. Following extensive washing, membranes were incubated with goat anti-mouse IgG (H+L)-HRP-conjugated antibody (Bio-Rad) at a 1:3000 dilution for 1 h at room temperature and immunoreactive proteins detected by enhanced chemiluminescence. Western blot images were captured using a G:BOX Bio-Imaging System and GeneSnap software (Syngene, Cambridge, UK). Equal loading of protein samples was verified with an anti-actin monoclonal antibody (Sigma-Aldrich). A parallel blot was performed using an isotype control antibody to exclude non-specific binding.

### Fluorescescence microscopy

To determine the subcellular location of PCLP1, cells were plated on 35 mm glass-bottom dishes (Ibidi, Martinsried, Germany) and stained with Hoechst 33342 (Thermo Fisher Scientific) to visualize the nuclei. For γ-tubulin staining, cells were plated on poli-D-lysine coated coverslips, fixed in methanol at −20°C for 10 min and blocked in PBS containing 1% FBS and 0.1% Triton X-100 for 30 min at room temperature. Afterwards, cells were incubated with anti-γ-tubulin mAb (BioLegend) at 1:500 dilution overnight at 4°C, followed by an incubation with Alexa Fluor 546-conjugated anti-mouse IgG antibody (Thermo Fisher Scientific) at 1:500 dilution for 1 h. Finally, cells were mounted on slides using mounting medium containing DAPI (Abcam, Cambridge, UK). Images were captured using an AxioCam MRm camera coupled to a Zeiss Observer Z1 fluorescence microscope equipped with an ApoTome.2 module and with a 63× oil immersion objective and ZEN 2 Pro software (Zeiss, Oberkochen, Germany).

To visualize lipid droplets, cells were plated on poli-D-lysine coated coverslips, fixed in 3.7% paraformaldehyde for 30 min at 4°C, stained with 40 µg/ml Nile Red (MP Biomedicals, Santa Ana, California, USA) and then mounted on slides using mounting medium containing DAPI (Abcam). Cells were examined using a Plan-Apochromat 60×/1.40 NA oil-immersion objective in an Eclipse TE2000-E inverted fluorescence microscope. For lipid droplet quantification, ten fields were randomly selected and images were captured with an ORCA-ER monochrome cooled-CCD camera and NIS-Elements AR imaging software. Then, the total area of lipid droplets per cell was calculated using ImageJ software (National Institute of Health) and the following formula: total lipid droplet area per field/cell number per field.

### Statistical analysis

Data are expressed as mean values ± standard deviation of the mean (mean ± SD). Statistical analysis was performed using Prism 3.0 Software (GraphPad) and SPSS 23. Statistical significance was calculated by two-tailed, paired Student´s *t*-test, no-paired Student´s *t*-test or Mann-Whitney *U* test. Values lower than 0.05 were considered significantly different (^*^p < 0.05; ^**^p < 0.01; ^***^p < 0.001).

## SUPPLEMENTARY MATERIALS FIGURES



## Abbreviations

2DG: 2-deoxy-D-glucose
6AN: 6-aminonicotinamide
7AAD: 7-aminoactinomycin D
B-ALL: B-cell acute lymphoblastic leukemia
BCR/BTK: B-cell receptor/Bruton´s tyrosine kinase
BMI-1: B-cell-specific lymphoma Moloney murine leukemia virus integration site 1 homolog
BL: Burkitt lymphoma
BSA: bovine serum albumin
CLL: chronic lymphocytic leukemia/small lymphocytic lymphoma
CXCL12: C-X-C motif chemokine 12
CXCR4: C-X-C chemokine receptor type 4
DLBCL: diffuse large B-cell lymphoma
FASN: fatty acid synthase
FBS: fetal bovine serum
FL: follicular lymphoma
G6PD: glucose 6-phosphate dehydrogenase
GFP: green fluorescence protein
GLS1: glutaminase 1
HCL: hairy cell leukemia
ICAM-1: intercellular adhesion molecule-1
LFA-1: leukocyte function-associated antigen-1
mAb: monoclonal antibody
MAPK: mitogen-activated protein kinase
MCL: mantle cell lymphoma
MFI: median fluorescence intensity
MZL: marginal zone B-cell lymphoma
PBMCs: peripheral blood mononuclear cells
PCLP1: podocalyxin
PI3K: phosphoinositide-3 kinase
PPP: pentose phosphate pathway
WM: lymphoplasmacytic lymphoma/Waldenström macroglobulinemia
