# Supplementary material for: Podocalyxin promotes proliferation and survival in mature B-cell non-Hodgkin lymphoma cells
Source: Oncotarget. 2017 Sep 27;8(59):99722–39. doi: 10.18632/oncotarget.21283 (PMC5725127; doi:10.18632/oncotarget.21283)
Supplement: Supplementary file 1 [file oncotarget-08-99722-s001.pdf]

# Podocalyxin promotes proliferation and survival in mature B-cell non-Hodgkin lymphoma cells

## SUPPLEMENTARY MATERIALS

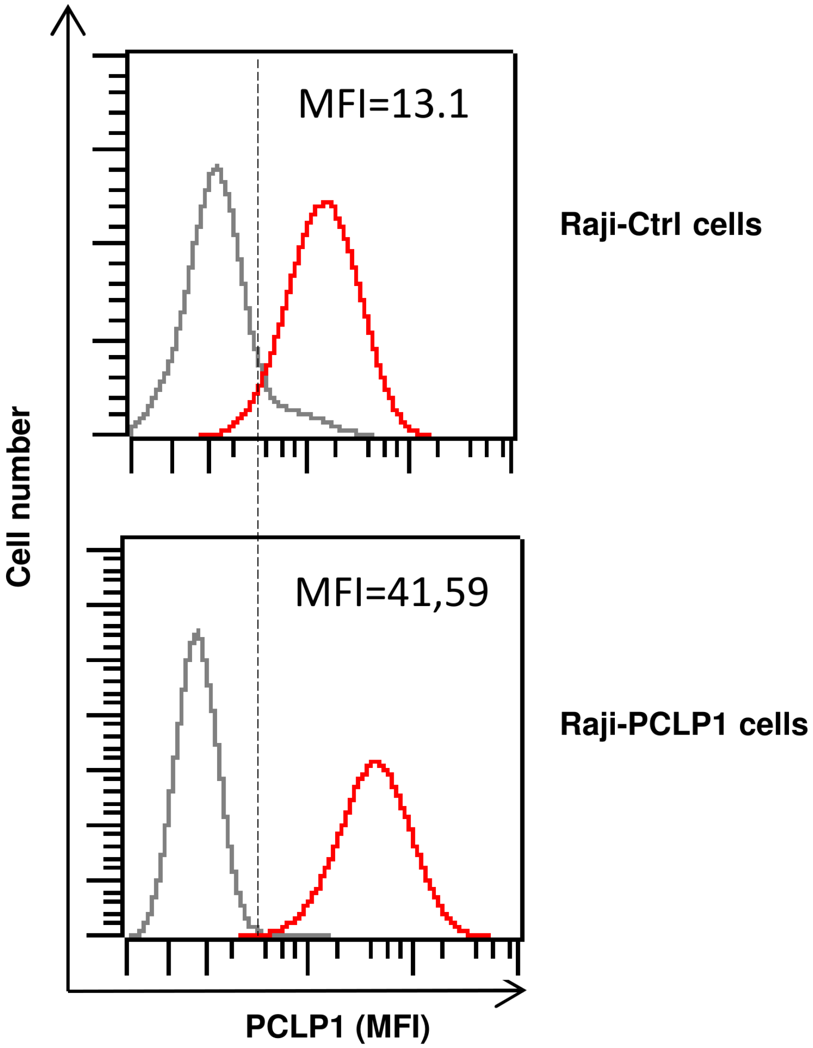


**Supplementary Figure 1: PCLP1 expression on the surface of Raji-PCLP1 cells.** Raji-PCLP1 and Raji-Ctrl cells were stained with an anti-PCLP1 monoclonal antibody conjugated with phycoerythrin and analyzed by flow cytometry. The red line and the grey line in the histograms represent PCLP1 staining with an anti-PCLP1 mAb and an isotype control, respectively. PCLP1 expression detected on Raji-Ctrl cells corresponds to endogenous PCLP1 and that detected on Raji-PCLP1 cells corresponds to endogenous plus ectopic (overexpressed) PCLP1. The median fluorescence intensity (MFI) of PCLP1 expression after isotype control subtraction is shown.

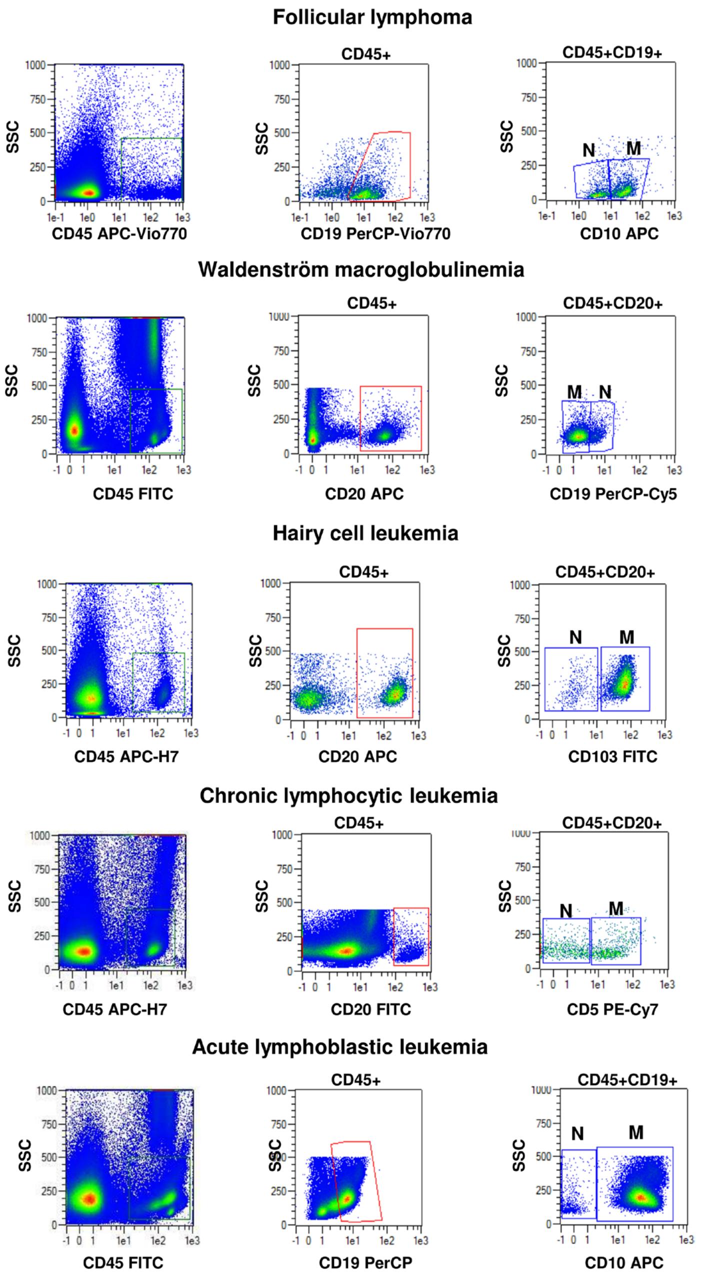


**Supplementary Figure 2: Multiparametric flow cytometry gating strategy for identification of malignant and normal B cells from B-cell lymphoma and B-cell ALL patients.** Cells from all subtypes of lymphoma were first gated based on CD45 positivity and side scatter characteristics using the CD45 vs SSC dot plot. Next, depending on the lymphoma subtype analyzed, different sequential gating strategies were applied to define malignant (M) and normal (N) cells as follows. In follicular lymphoma cases, B cells were identified based on CD19 expression of CD45-gated cells. Then, normal and malignant cells were detected based on CD10 expression of CD19 positive population. In Waldenström macroglobulinemia, B cells were identified based on CD20 positivity of CD45-gated cells and next, normal and malignant cells were selected according to CD19 expression level of CD20 positive cells. In hairy cell leukemia, normal and malignant cells were defined based on CD103 expression of CD20 positive B cells. In chronic lymphocytic leukemia, normal and malignant cells were identified based on CD5 expression of CD20 positive B cells. Finally, normal and malignant cells from acute lymphoblastic leukemia were selected based on CD10 expression of CD19 positive cell population.

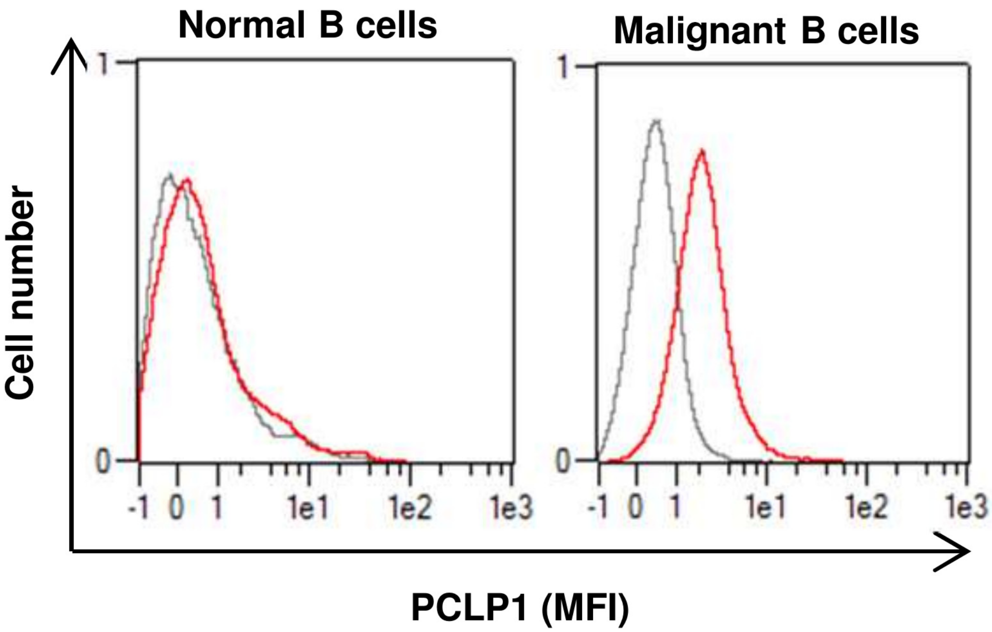


**Supplementary Figure 3: PCLP1 expression in mature B-cell lymphomas.** Representative flow cytometry histograms showing PCLP1 expression on normal and malignant B cells from a patient with lymphoma are shown. The red line and the grey line in the histograms represent PCLP1 staining with an anti-PCLP1 mAb and an isotype control, respectively.

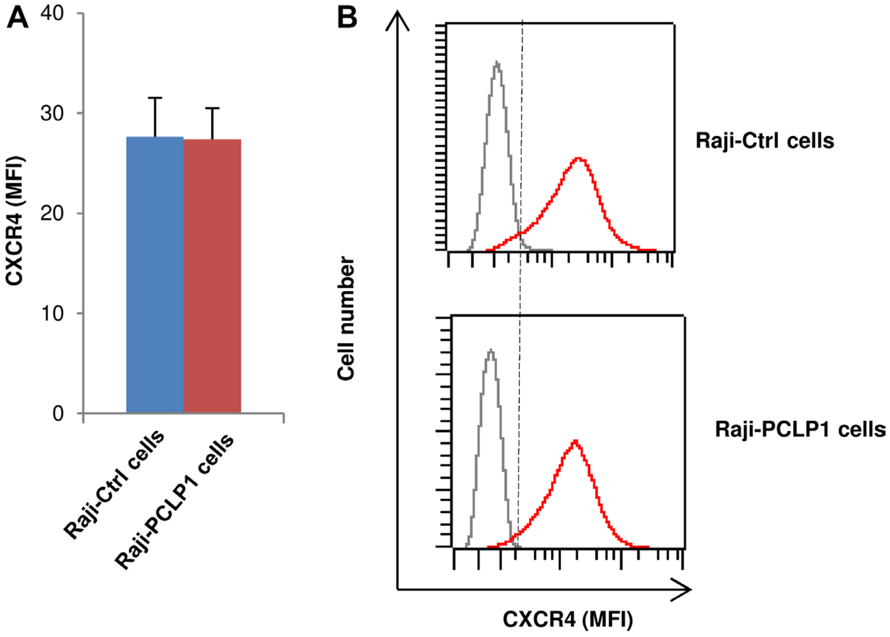


**Supplementary Figure 4: CXCR4 expression on the surface of Raji cells overexpressing PCLP1.** Raji-PCLP1 and Raji-Ctrl cells were stained with an anti-CXCR4 monoclonal antibody conjugated with APC and analyzed by flow cytometry. (**A**) Graph shows MFI of CXCR4 expression from four independent experiments. Data represent mean ± SD. (**B**) Representative histograms of flow cytometry showing CXCR4 expression on Raji-PCLP1 and Raji-Ctrl cells. The red line and the grey line in the histograms represent CXCR4 staining with an anti-CXCR4 mAb and an isotype control, respectively.

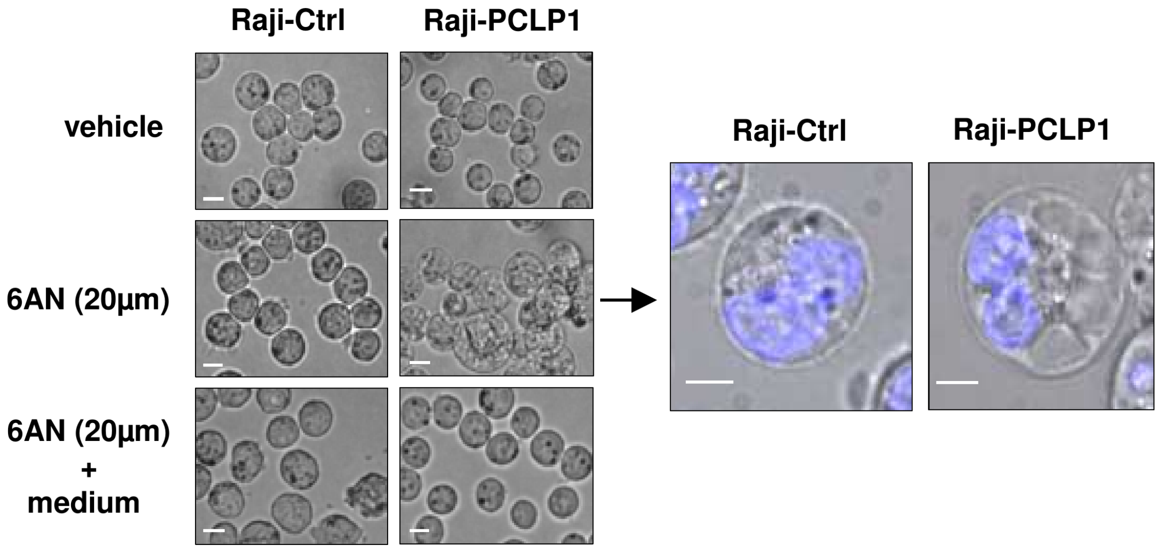


**Supplementary Figure 5: The PPP inhibitor 6AN induces the vacuolation of Raji-PCLP1 cells.** Raji-Ctrl and Raji-PCLP1 cells were cultured in the presence of vehicle (upper panels) or 6AN, an inhibitor of PPP (middle panels) for 96 h and observed by bright field microscopy. Then, the medium containing 6AN was removed and cells were further cultured for 96 h in fresh medium without the inhibitor (lower panels). The size bar indicates 10 µm. An amplified image of Raji-Ctrl and Raji-PCLP1 cells treated with 6AN is depicted to show the vacuolization of 6AN-treated Raji-PCLP1 cells. Nuclei were stained with Hoechst 33342 (blue). The size bar indicates 5 µm.
